# Increased Soluble CD4 in Serum of Rheumatoid Arthritis Patients Is Generated by Matrix Metalloproteinase (MMP)-Like Proteinases

**DOI:** 10.1371/journal.pone.0063963

**Published:** 2013-05-21

**Authors:** Wen-Yi Tseng, Yi-Shu Huang, Nien-Yi Chiang, Yeh-Pin Chou, Yeong-Jian Jan Wu, Shue-Fen Luo, Chang-Fu Kuo, Ko-Ming Lin, Hsi-Hsien Lin

**Affiliations:** 1 Division of Rheumatology, Allergy and Immunology, Chang Gung Memorial Hospital at Keelung, Keelung, Taiwan; 2 Graduate Institute of Clinical Medicine, Chang Gung University, Tao-Yuan, Taiwan; 3 Graduate Institute of Biomedical Sciences, Chang Gung University, Tao-Yuan, Taiwan; 4 Department of Medicine, College of Medicine, Chang Gung University, Tao-Yuan, Taiwan; 5 Division of Hepato-Gastroenterology, Department of Internal Medicine, Chang Gung Memorial Hospital at Kaohsiung, Kaohsiung, Taiwan; 6 Division of Rheumatology, Allergy and Immunology, Chang Gung Memorial Hospital at Linkou, Linkou, Taiwan; 7 Division of Rheumatology, Allergy and Immunology, Chang Gung Memorial Hospital at Chiayi, Chiayi, Taiwan; 8 Department of Microbiology and Immunology, College of Medicine, Chang Gung University, Tao-Yuan, Taiwan; University of Leuven, Rega Institute, Belgium

## Abstract

Higher soluble CD4 (sCD4) levels in serum have been detected in patients of infectious and chronic inflammatory diseases. However, how and why sCD4 is produced remains poorly understood. We establish sensitive ELISA and WB assays for sCD4 detection in conditioned medium of *in vitro* cell culture system and serum of chronic inflammatory patients. Serum samples from patients with systemic lupus erythematosus (SLE) (n = 79), rheumatoid arthritis (RA) (n = 59), ankylosing spondylitis (AS) (n = 25), gout (n = 31), and normal controls (n = 99) were analyzed using ELISA for sCD4 detection. Results from each assay were analyzed by the Kruskal-Wallis test. Dunn’s multiple comparison post-test was then applied between groups. We confirm that cells expressing exogenous CD4 produce sCD4 in a constitutive and PMA-induced manner. Importantly, sCD4 production in a heterologous expression system is inhibited by GM6001 and TAPI-0, suggesting receptor shedding by matrix metalloproteinase (MMP)-like proteinases. Moreover, similar findings are recapitulated in human primary CD4^+^ T cells. Finally, we show that serum sCD4 levels are increased in patients of chronic inflammatory diseases including RA and SLE, but not in those with gout. Intriguingly, sCD4 levels in RA patients are correlated positively with the disease activities and higher sCD4 levels seem to associate with poor prognosis. Taken together, we conclude that CD4 is shed from cell surface by a MMP-like sheddase and sCD4 level is closely related with the inflammatory condition in certain chronic diseases. Hence, sCD4 might be considered an important parameter for RA disease progression with potential diagnostic importance.

## Introduction

CD4 is a 60-kDa glycoprotein of the immunoglobulin superfamily (IgSF), containing four extracellular Ig-like domains, a hydrophobic transmembrane region and a 40-residue cytoplasmic tail [1]. CD4 is expressed in many immune cells including T cells, monocytes, macrophages and dendritic cells. The role of CD4 in T cells is multifaceted so that it is involved in T cell differentiation and development as well as T cell activation by interacting with antigen-presenting cells (APCs). The cytoplasmic tail of CD4 associates with the Lck kinase, which in turn activates the signaling components of the T cell receptor (TCR)-CD3 complexes [2], [3], [4]. As such, one of the major functions of CD4 is to augment the TCR signaling during T cell-APC interaction by acting as a co-receptor.

In fact, due to its functional significance as a co-receptor, CD4 has been clinically tried as a major target in T cell-targeted therapies for the treatment of T cell-mediated autoimmune diseases such as rheumatoid arthritis (RA). Indeed, CD4-specific monoclonal antibodies (mAb) were among the first biologic therapies developed for rheumatic diseases [5]. Both depleting and non-depleting mAbs against CD4 have been administrated in RA patients in an attempt to interrupt T cell functions, but were determined to be ineffective in randomized clinical trials [6], [7]. The underlying mechanisms for the unfavorable clinical outcome following CD4 mAb treatment are multifaceted and complex [6], [7].

CD4 also is the high-affinity entry receptor for human immunodeficiency virus (HIV) by binding to the viral envelope glycoprotein gp120 [8]. HIV apparently escapes the effect of neutralizing antibodies by generating new variants, but infection of T cells still requires gp120-CD4 interaction. Therefore, one approach to block HIV infection is to use the soluble form of CD4 (sCD4) to inhibit virus attachment to target cells. Indeed, recombinant sCD4 was shown effective in blocking HIV binding to CD4^+^ T cells *in vitro*, and hence was considered a potential target for anti-HIV therapy [9], [10].

Interestingly, elevated serum sCD4 has been found in patients of viral infections, such as HIV [11] and Epstein-Barr virus [12]. In addition, serum sCD4 was also identified in patients of chronic inflammatory diseases such as RA [13], Sjogren’s syndrome [14], systemic lupus erythematosus (SLE) [14], common variable immunodeficiency [15], osteoarthritis (OA) [13], chronic renal failure [16], and localized scleroderma [17]. In prospective sequential studies of RA patients, serum sCD4 levels were found to correlate positively with the clinical disease status [13]. Therefore, unveiling the mechanism of sCD4 generation is of important relevance to understanding the role of CD4 receptor in these diseases. Delineation of the relationship between sCD4 and cellular CD4 receptor might also help explain the ineffective effect of CD4 mAbs in the RA clinical trials.

Soluble forms of transmembrane proteins, including adhesion molecules and receptors, which still retain biological activities have been identified in various extracellular compartments. At least three independent cellular processes are known to be involved in their production: First, soluble cytokine receptors can be generated by alternative splicing of mRNA transcripts; Second, cytokines and soluble cytokine receptors can be released as membrane components of cellular vesicles such as exosomes that are small membrane-enclosed entities (typically <100 nm in diameter); Third, extracellular proteinases such as matrix metalloproteinases (MMPs) actively target and shed the extracellular domain of cell surface proteins [18], [19].

Previous studies have failed to detect any CD4 transcripts with deletion/premature termination at/around the transmembrane region, suggesting that sCD4 is unlikely resulted from alternatively-spliced mRNAs [20]. Through the use of flow cytometry, ELISA, and Western blotting analyses in primary CD4^+^ T cells and cells expressing exogenous CD4, herein we unequivocally show that sCD4 is produced mainly via receptor shedding by MMP-like proteinases. Moreover, we found that serum sCD4 levels in chronic inflammatory diseases such as SLE and RA, are strongly elevated. Most significantly, serum sCD4 levels of RA patients are positively correlated with the disease status defined by the 28-joint count disease activity score (DAS28). Hence, we conclude that CD4 shedding is predominantly mediated by MMPs and abnormal expression and activity of MMPs in certain chronic inflammatory diseases may enhance serum sCD4 levels, which can be considered as a potential diagnostic parameter for chronic inflammatory disease progression.

## Materials and Methods

### Reagents, Antibodies and Cell Culture

Unless otherwise specified, general reagents and antibodies (Abs) were obtained from Sigma-Aldrich (St. Louis, MO, USA). DNA and protein reagents were obtained from Clontech (CA, USA), Invitrogen (Carlsbad, CA), Qiagen (Valencia, CA), Fermentas (ON, Canada) or New England Biolabs (MA, USA). Monoclonal Abs (mAbs) used in the study are: EMR2 stalk-specific 2A1 and anti-CD4 (1F6) from AbD Serotec (Kidlington, UK); anti-CD4 (OKT4) and biotin-conjugated anti-CD4 (RPA-T4) from eBioscience (San Diego, CA, USA); Anti-c-myc (9E10) from Invitrogen; PE-conjugated anti-CD4, anti-CD62L, anti-CD69 and mouse IgG_1_ isotype control were from BD Systems (MN, USA). Cell culture media and supplements including 10% heat inactivated fetal calf serum (FCS), 2 mM L-glutamine, 50 IU/ml penicillin and 50 µg/ml streptomycin were purchased from Invitrogen. CHO-K1 and primary CD4^+^ T cells were cultured in Ham’s F-12 and RPMI medium, respectively.

### Human CD4 Expression Construct

The human full-length CD4 expression construct, hCD4-myc, was generated using the full-length CD4 cDNA PCR-amplified from pooled human peripheral leukocytes total RNA (BD Biosciences-Clontech), and subcloned into the *Hin*dIII-*Xho*I sites of pcDNA3.1myc-His C (Invitrogen). The fidelity of the coding sequence was confirmed by DNA sequencing.

### Patients

This study was approved by the Chang Gung Memorial Hospital Ethics Committee (CGMF IRB No.: 97-1457B and 98-2805B) and all procedures were performed according to the guideline set by the Committee. The number of patients recruited for this study include: systemic lupus erythematosus (SLE), 79; rheumatoid arthritis (RA), 59; Ankylosing spondylitis (AS), 25; and gout, 31. A total of ninety-nine healthy volunteers are also included. All participants were recruited from the outpatient clinics of Chang Gung Memorial Hospital (Taiwan) from 2008 to 2010. Patients were screened to meet the criteria set by American College of Rheumatology for the diagnosis of SLE, RA or gout, and the modified New York criteria for AS. All were evaluated by specific validated disease activity indexes such as Systemic Lupus Erythematosus Activity Index (SLEDAI) (median: 4, interquartile range: 2–8), DAS28 (median:4.55, interquartile range: 2.90–5.85), Bath Ankylosing Spondylitis Disease Activity Index (BASDAI) (median: 2.7, interquartile range: 1.9–5.4) or Bath Ankylosing Spondylitis Functional Index (BASFI) (median: 1.55, interquartile range: 0.59–5.425) at the outpatient clinic from signs and symptoms as well as the results of laboratory tests. All gout patients recruited in this study had at least one episode of acute gouty arthritis attack in past one week. At the time of inclusion, 59 of 79 SLE patients were being treated with corticosteroids (prednisolone 2.5–30 mg/day). Almost all RA patients were already receiving at least one disease modifying anti-rheumatic drug and coricosteroids at inclusion (38 patients with MTX 5 mg-15 mg/week, 7 patients with leflulomide 10–20 mg/day, 31 patients with sulfasalazine 1–2 gm/day, 40 patients with hydroxycholroquine 200–400 mg/day and 44 patients with prednisolone 5–15 mg/day) and part of them also receiving biologic agents (16 patient with tumor necrosis blockers and 1 patients with rituximab). Almost all AS patients were treated with non-steroid anti-inflammatory drugs and some of them were treated with sulfasalazine (17 patients with sulfasalazine 1–2 gm/day) at inclusion. Patients and healthy volunteers were informed about the nature of the experimental procedures and all have signed written informed consent. Serum samples (∼10 ml) were taken from patients, prepared as described previously [21] and stored at −80°C until use.

### Transient Transfection and Drug Treatment

Transient transfection of expression constructs was performed using Lipofectamine™ (Invitrogen) as described previously [22]. Transfected cells were sub-cultured into 6-well plate and fed with fresh medium for 1–2 day for further drug treatment. Cells were cultured for 2 days in OPTI-MEM containing protease inhibitors (CALBIOCHEM, CA, USA) such as GM6001 (50 µM), E-64 (50 µM), Pepstatin A (50 µM), α_2_ macroglobulin (50 µM), Furin protease inhibitor I (50 µM), TAPI-0 (10 µM), TAPI-1(10 µM), or TAPI-2 (10 µM). When necessary, cells were also treated with 12-*O*-tetradecanoylphorbol- 13-acetate (PMA) in OPTI-MEM as indicated. In addition, CD4^+^ T cells were treated with PMA in serum-free RPMI medium.

### Immunoblotting Analysis

Cells were lysed in RIPA lysis buffer (20 mM TrisHCl pH7.4, 5 mM MgCl_2**,**_ 100 mM NaCl, 0.5% NP-40 and 1X Complete Protease Inhibitors) supplemented with 1 mM sodium orthovanadate, 1 mM AEBSF and 5 mM Levamisole. Proteins were quantified using the Bicinchoninic acid (BCA) protein assay kit (PIERCE, Rockford, USA). Conditioned medium (CM) of CHO-K1 cells transfected with hCD4FL-myc was collected and concentrated using Amicon Ultra centrifugal filters-10 kDa cut-off (Millipore, MA, USA). SDS-PAGE and western blot (WB) analyses were carried out using standard procedures as described previously [22], [23].

### Flow Cytometry

After treatment, primary CD4^+^ T cells were washed, and fixed with fresh prepared 2% paraformaldehyde solution in PBS at 4°C for 30 min. Cells were then blocked for 1 hr in ice-cold blocking buffer (PBS buffer containing 1% BSA/5% normal serum of the animal where the secondary Ab was derived from). Cells were subsequently incubated with the indicated primary Ab diluted in blocking buffer for 1 hr, and then washed three times by cold PBS and subjected to analysis by FACScan flow cytometer (BD Biosciences).

### Detection of Soluble CD4 by ELISA

To analyze the concentration of soluble CD4 (sCD4) in serum and culture conditioned medium, an ELISA assay utilizing two noncompeting murine mAbs to the human CD4 protein are established in house. Briefly, anti-CD4 mAb (clone: OKT4; 2.5 µg/ml) was diluted in PBS and coated onto the 96 well plate (100 µl/well) by incubating at 37°C for 2 h. The mAb solution was then discarded and the wells incubated with blocking buffer (1% bovine serum albumin (BSA) in PBS) overnight at 4°C. Following extensive washes with PBS/0.1% (v/v) Tween-20, samples (100 µl/well) were added and incubated at RT for 1 h. Recombinant sCD4 proteins (∼7–2000 pg/ml) diluted in blocking buffer were used as standard. After discarding samples, wells were extensively washed and 100 µl of biotin-conjugated second anti-CD4 mAb (clone: RPA-T4; 0.5 µg/ml) was added for incubation at RT for 1 h. Wells were again washed extensively and then incubated with 100 µl of avidin-HRP (Sigma-Aldrich) (1∶200 diluted in blocking buffer) for 1 hr at RT. Finally, wells were washed and incubated with 100 µl of O-phenylenediamine (0.5 mg/ml) in citrate-phosphate buffer containing 0.01% (v/v) hydrogen peroxide (BD Biosciences) for 20 min at RT in the dark. The reaction was stopped by adding 50 µl of 2 N H_2_SO_4_ per well. The 450 nm OD reading of the plate was done in a Dynatech MR700 microplate reader (Tecan, Switzerland).

### CD4^+^ T Cell Purification

Peripheral blood monocytic cells (PBMCs) were isolated from fresh venous blood of healthy donors using a protocol approved by the institutional review board at Chang Gung Memorial Hospital (CGMF IRB No.: 97–1457B and 98–2805B). Written informed consent was signed by all participants. In short, fresh blood was collected and spun at 2000 rpm for 20 min at RT. Blood plasma at the upper fraction was discarded, while blood cells at the lower fraction were resuspended with equal volume of 1X PBS. Blood cells were overlaid onto a half volume of Ficoll Hypaque (Chalfont St Giles, UK), taking care not to destroy the interface between the two fractions. Cells were centrifuged at 2000 rpm for 20 min at RT, resulting in the generation of the PBMC fraction between serum and Ficoll Hypaque fractions for collection. Subsequently, CD4^+^ T cells were isolated from PBMCs by CD4^+^ T cell isolation kit ΙΙ (MACS, Germany) according to the manufacturer’s protocols. In brief, PBMCs were washed twice, counted, and resuspended in 2∼5 mM cold EDTA buffer at the cell density of 1×10^7^ cells/40 µl. Cells were mixed with 10 µl of biotin-Ab cocktail and incubated for 10 min at 4°C, followed by the addition of 30 µl of 2∼5 mM cold EDTA buffer and 20 µl of anti-biotin microbeads. The cell-Ab-microbead reaction mixture was incubated for 15 min at 4°C. Cells were then washed thoroughly with 1X cold PBS. Cells were resuspended in 1 ml of 2.5 mM cold EDTA buffer for CD4^+^ T cell purification using the LS (MACS) column.

### Statistical Analysis

The measured items and domain scores of the five study groups were presented as mean and standard deviation. All statistical tests were performed with the use of SPSS-19 software (SPSS Inc., USA). The ELISA data of *in vitro* cell culture systems and serum samples of patients did not fit a Gaussian distribution despite attempts at log transformation. Non-parametric Kruskal-Wallis test was therefore used analyze these data to determine if there was significant variation in the medians of the groups analyzed. If 95% significance was achieved, Dunn’s multiple comparison post-test was then used to compare the assay results of one group with another. Correlations between sCD4 and gender, age and disease activity index of SLE (SLEDAI) and RA (DAS28) were analyzed according to Spearman’s rank correlation coefficient. A trend analysis was used to compare the proportions of different kinds of outcomes between low level sCD4 (<0.125 ng/ml) and high level sCD4 (≧0.125 ng/ml) groups in RA patients, as well as the dose- and time-dependent response of PMA treatment. In all cases, a p-value of <0.05 (two sided) was considered statistically significant. *P*-values are as follows: ^*^ *P*<0.05, ^**^ *P*<0.01 and ^***^ *P*<0.001. The ELISA data of *in vitro* cell culture systems are means ± S.E.M. of 3 independent experiments performed in triplicate.

## Results

### Evidence that Soluble CD4 is Generated via Receptor Shedding

**Figure 1:**
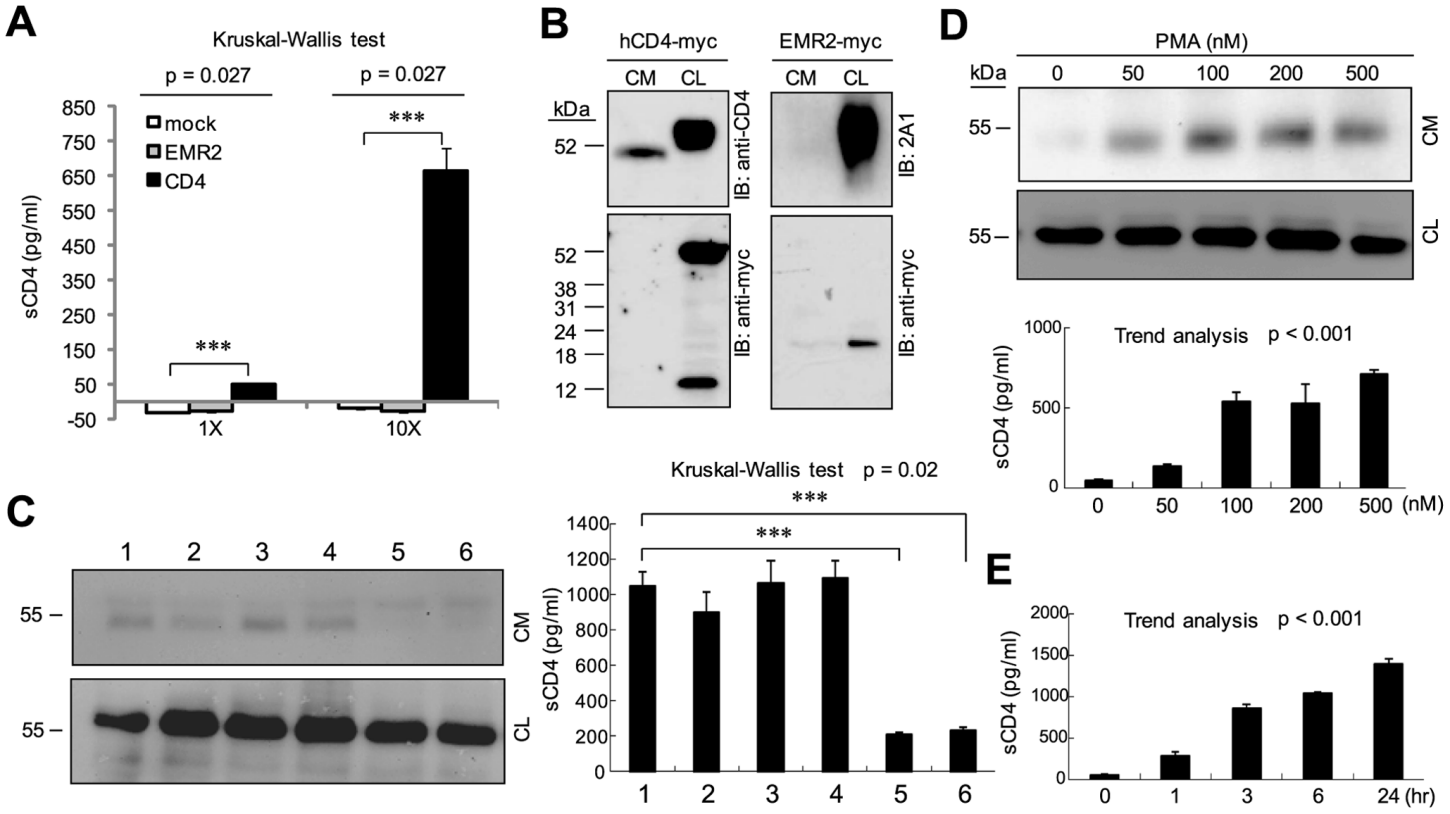
CD4 is shed by MMP-like proteinases in transfected CHO-K1 cells. (A, B) CHO-K1 cells were transiently transfected with the hCD4-myc or negative control EMR2-myc expression construct. Production of sCD4 is evaluated by ELISA (A) and WB (B) analysis. Conditioned medium (CM) and cell lysates (CL) were probed with 2A1 (specific to EMR2), anti-CD4 and anti-myc mAbs in WB analysis. Kruskal-Wallis test across all three groups of 1X and 10X concentration shows p = 0.027. Dunn’s multiple comparisons post-test p values between groups show ***P<0.001. (C) sCD4 production is inhibited by pan-MMP inhibitors, GM6001 and TAPI-0. CHO-K1 cells transiently transfected with the hCD4-myc construct were treated with various protease inhibitors (1, Control; 2, DMSO; 3, Furin protease inhibitor I; 4, Pepstatin A; 5, GM6001; 6, TAPI-0) then analyzed by WB (left panel) and ELISA (right panel). Kruskal-Wallis test across all six groups shows p = 0.02. Dunn’s multiple comparisons post-test p values between groups show ***P<0.001. (D, E) CHO-K1 cells transiently transfected with the hCD4-myc construct were treated with PMA in various concentrations (D) for 1 hr or 50 nM PMA for different time periods (E) then analyzed by ELISA and WB. ELISA. Statistical significance was assessed by the trend analysis.

To date the molecular mechanism(s) whereby soluble CD4 (sCD4) is generated remains unclear. Previous studies have examined, but failed to detect novel CD4 RNA transcripts with deletion or premature termination signals at/before the transmembrane region. This suggested that RNA alternative splicing is unlikely the main cause for sCD4 [20]. Other alternatives include receptor exocytosis and shedding. To systematically investigate the potential mechanism(s) involved, we first established a sensitive sandwich ELISA assay to detect sCD4 in a heterologous cell expression system. Indeed, sCD4 can be readily detected by the ELISA assay in the conditioned medium (CM) of CHO-K1 cells expressing the full-length human CD4 with a C-terminal myc tag (hCD4-myc) (Fig. 1A). No signal was detected in the CM of mock-transfected cells or cells expressing an unrelated EMR2 receptor (Fig. 1A).

Similarly, WB analysis identified sCD4 in the CM of hCD4-myc expressing cells, but no signal in the CM of cells expressing human EMR2, indicating the specific expression and generation of sCD4 (Fig. 1B). Most interestingly, the size of the sCD4 band found in CM (∼50 kDa) is smaller than that detected in the whole cell lyaste (CL) (∼60 kDa), suggesting a possible shedding event. This idea is further strengthened by the fact that apart from the ∼60 kDa full-length CD4 band, a ∼13 kDa fragment is detected in the CL by anti-myc mAb (Fig. 1B and Fig. S1, S2). The ∼13 kDa fragment most likely represents the C-terminal half of CD4 receptor including the transmembrane region and cytoplasmic tail. Hence, we conclude that a portion of the surface CD4 receptor is likely modified by ectodomain shedding to produce a smaller sCD4.

### CD4 Shedding is Mediated by Metalloproteinases (MMPs)

To identify the candidate sheddase(s) involved in the ectodomain shedding of CD4, transiently-transfected CHO-K1 cells were treated with various protease inhibitors. WB analysis shows that while most protease inhibitors show no apparent effect on CD4 shedding, GM6001 and TAPI-0 efficiently diminished the production of sCD4 (Fig. 1C and Fig. S1, S2). This result strongly suggests that CD4 shedding is mediated by metalloproteinase(s) as both GM6001 and TAPI-0 are potent broad-spectrum hydroxamate inhibitors of metalloproteinases such as matrix metalloproteinases (MMPs) and members of a disintegrin and metalloprotease (ADAM) family [24]. To further verify this suggestion, cells were treated with 12-*O*-tetradecanoylphorbol- 13-acetate (PMA), which is well known to induce metalloproteinase activity [25]. As expected, PMA-treated CHO-K1 cells produce sCD4 in a dose- and time-dependent manner (Fig. 1D and E). Hence, we conclude that the ectodomain shedding of CD4 is mediated predominantly by MMPs.

### Primary CD4^+^ T cells also Produce sCD4 via MMP-like Mediated Ectodomain Shedding

**Figure 2:**
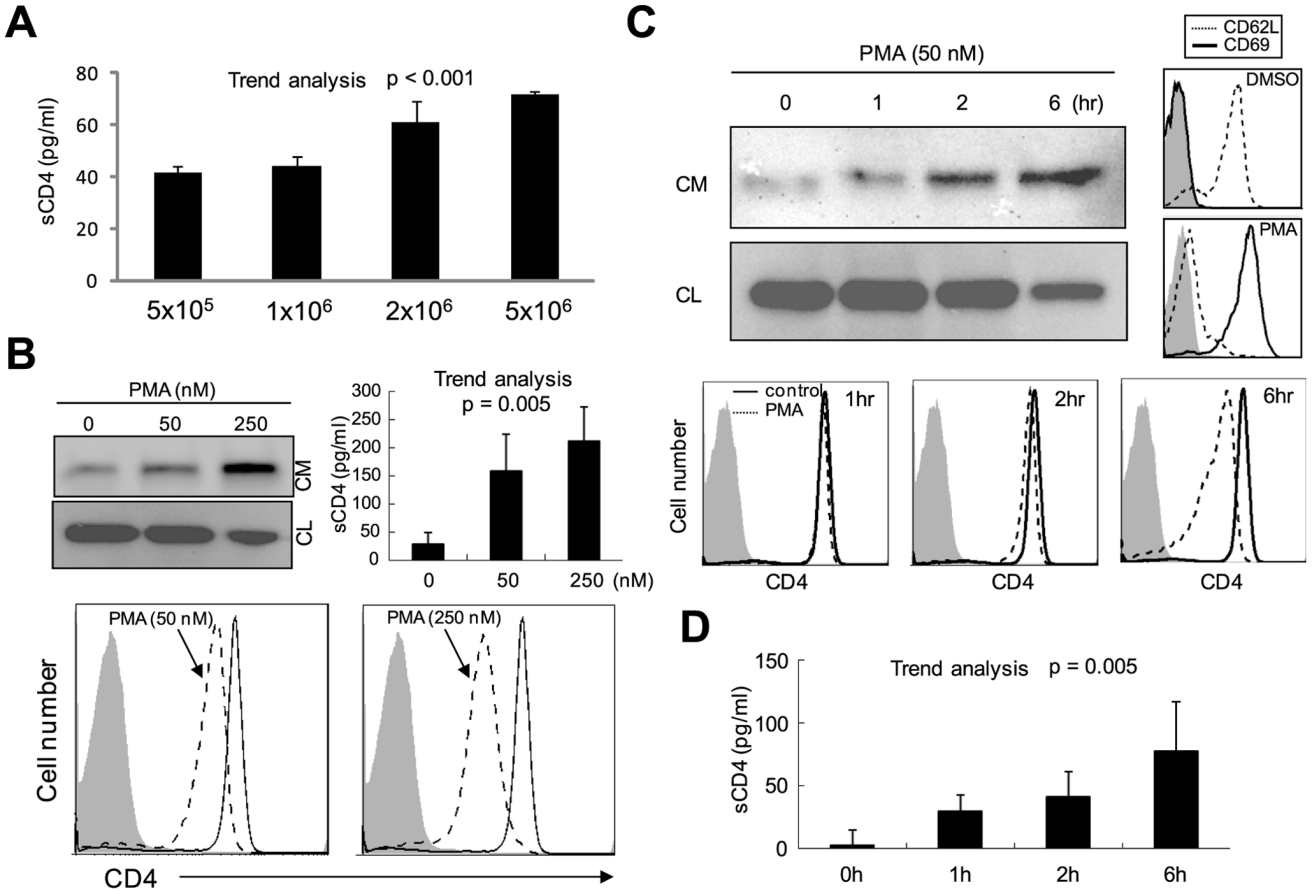
sCD4 is also generated in primary CD4^+^ T cells. (A) CD4^+^ T cells were purified from healthy volunteers and cultured in different cell densities as indicated. CM was then collected for ELISA analysis. (B-D) 1×10^6^ CD4^+^ T cells were stimulated by PMA in different doses (B) for 3 hr or 50 nM PMA for different time periods (C and D) before collecting CM, CL and cells for ELISA, WB and FACS analysis. In addition, stimulated CD4^+^ T cells were stained with cell activation markers CD62L and CD69 and analyzed by FACS analysis (C). Statistical significance was assessed by the trend analysis.

**Figure 3:**
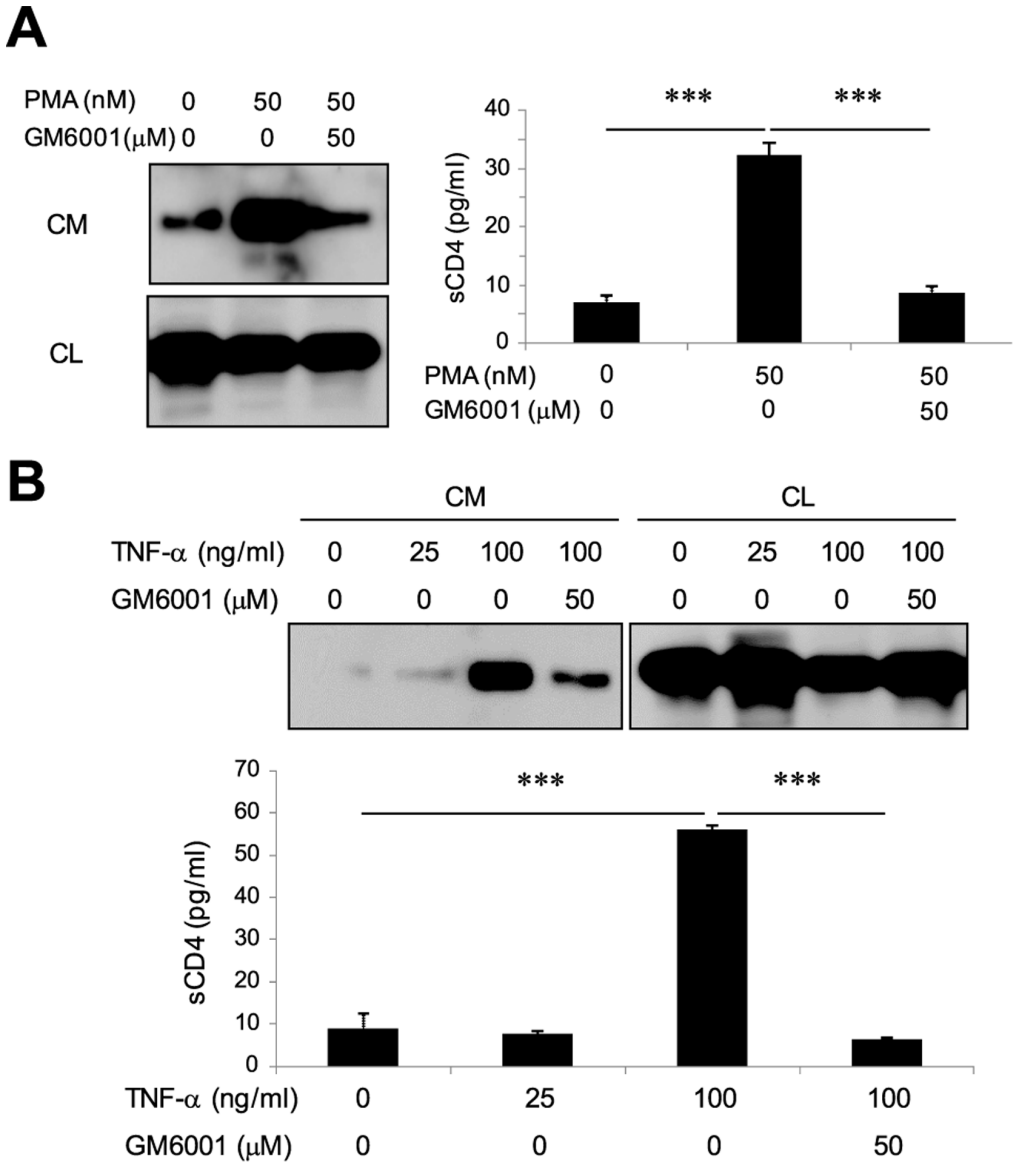
Production of sCD4 by primary CD4^+^ T cells is mediated by MMP-like shedding. (A, B) CD4^+^ T cells were stimulated by 50 nM PMA (A) or TNF-α (25 and 100 ng/ml) (B) in the absence or presence of GM6001 (50 µM) for 3 hr. CM and CL were collected for ELISA and WB analysis. Dunn’s multiple comparisons post-test p values between groups show ***P<0.001.

To examine whether human primary CD4^+^ T cells also generate sCD4 by a similar mechanism(s), CD4^+^ T cells were isolated for analysis. ELISA analysis indicates that sCD4 is generated constitutively in a cell density-dependent manner (Fig. 2A). In addition, PMA-treated CD4^+^ T cells enhances the generation of sCD4 in a dose- and time-dependent manner (Fig. 2B, C and D). Consequently, reduced surface CD4 levels were found in PMA-treated T cells by FACS analysis (Fig. 2B and C). During T cell activation, CD69 is highly up-regulated while ADAM17-mediated CD62L shedding is enhanced [26], [27], [28], [29], [30]. Therefore, PMA-induced T cell activation is confirmed by reduced and enhanced surface expression of T cell activation markers CD62L and CD69, respectively (Fig. 2C). Consistent with earlier results, WB and ELISA analyses showed that GM6001 treatment effectively inhibits PMA-induced CD4 shedding in T cells (Fig. 3A). Finally, sCD4 was detected in the CM of T cells activated by TNF-α in a dose-dependent manner that is inhibited by GM6001 (Fig. 3B). All together, these results indicate that CD4 receptor shedding occurs constitutively at a basal level in CD4^+^ T cells, and T cell activation enhances CD4 shedding that is sensitive to GM6001.

### Serum sCD4 Levels are Elevated in Patients of Autoimmune Diseases

**Figure 4:**
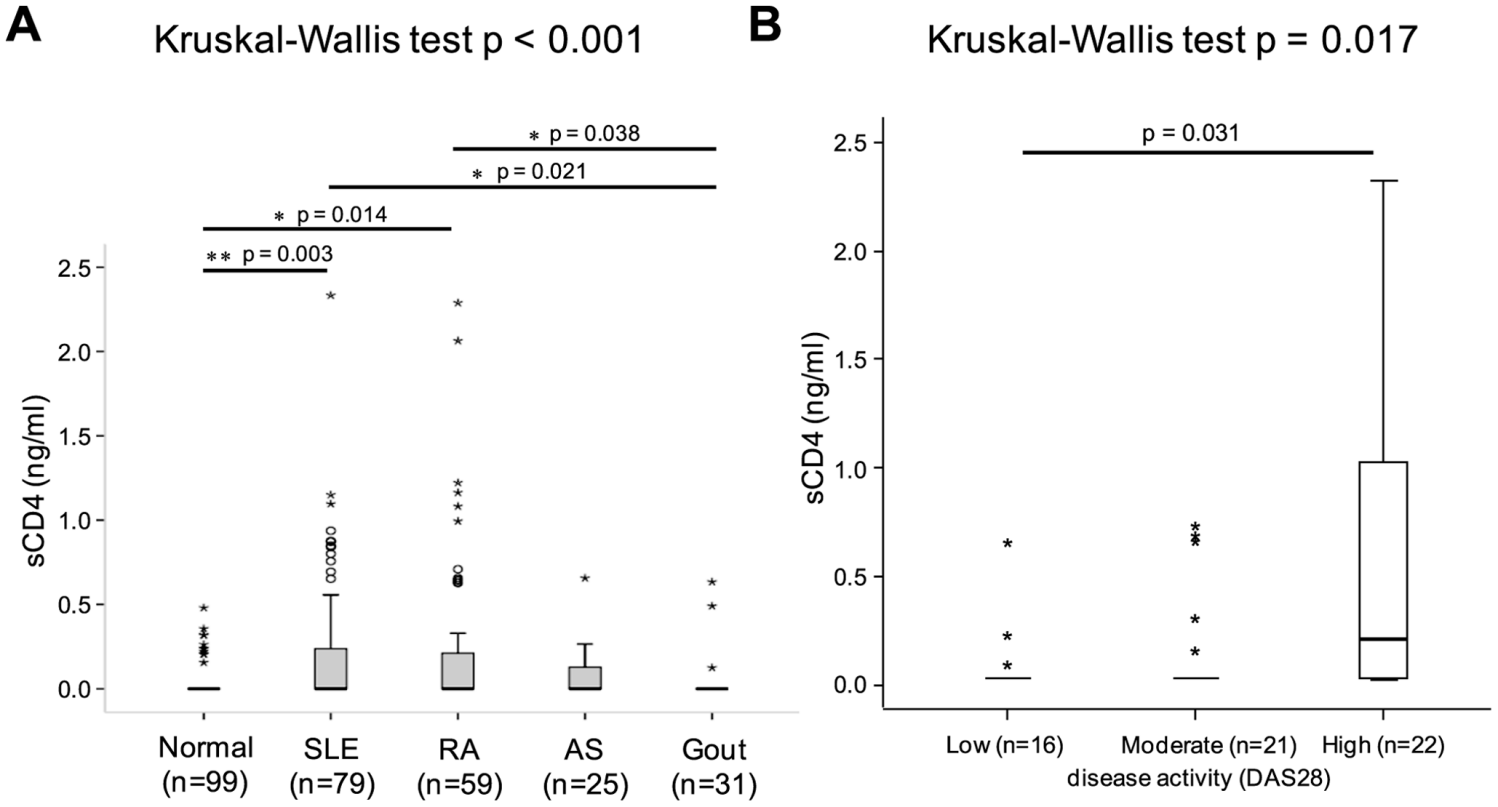
Comparison of serum sCD4 levels in various chronic inflammatory diseases. (A) Serum sCD4 levels in normal subjects groups, SLE, RA, AS, and gout. Data are shown as box plots. Each box represents the 25th to 75th percentiles. Lines inside the boxes represent the median. Lines outside the boxes represent the 10th and the 90th percentiles. Circles and asterisks indicate outliers and extreme values, respectively. Kruskal-Wallis test across all five groups shows p<0.001. Only significant Dunn’s multiple comparisons post-test p values between groups are shown. (B) Comparison of serum sCD4 levels in RA patient groups classified by DAS28 as low-disease, moderate-disease, and high-disease activity. Data are shown as box plots. Each box represents the 25th to 75th percentiles. Lines inside the boxes represent the median. Lines outside the boxes represent the 10th and the 90th percentiles. Asterisks indicate extreme values. Kruskal-Wallis test across all three groups shows p = 0.017. Only significant Dunn’s multiple comparisons post-test p values between groups are shown.

To determine whether the serum sCD4 level is relevant to disease progression, we screen patients of SLE, RA, and AS as these are well-known autoimmune diseases with typical chronic inflammation and leukocyte activation. Gout patients representing acute inflammation are also included for comparison. The concentrations of sCD4 in the serum of 194 patients with SLE, RA, AS and gout, as well as 99 normal subjects were assessed by the ELISA assay as described above. As shown in Fig. 4A and Table 1, patients with SLE and RA had significantly higher concentrations of sCD4 than the normal subjects. In the AS patient group, serum sCD4 levels were also elevated considerably though not statistically significant in comparison to controls. On the contrary, serum sCD4 level of 31 gout patients was not different from those of the normal control subjects.

**Table 1:** Clinical characteristics and sCD4 levels of normal donors and subjects with SLE, RA, AS and gout.

Variable	SLE (N = 79)	RA (N = 59)	AS (N = 25)	Gout (N = 30)	Normal donor (N = 99)
Age (y/o)	42.85±11.65	54.92±10.43	41.44±14.53	50.13±16.14	48.31±10.87
Male Sex no. (%)	7 (8.86)	8 (13.56)	18 (72)	30 (100)	82 (82.83)
Soluble CD4 (ng/ml) median (IQR 25^th^–75^th^)	0(0–0.272)	0(0–0.22)	0(0–0.134)	0(0–0)	0(0–0)

Data are expressed as means ± SD and median (Interquartile range, 25^th^–75^th^).

SLE, systemic lupus erythematous; RA, rheumatoid arthritis; AS, ankylosing spondylitis;

IQR, interquartile range.

The five patient groups differed significantly in sex and age when the demographic and clinical data are taken into consideration. However, an examination of the effect of sex on serum sCD4 levels in the controls showed no significant differences between men and women. Similarly, no correlation was found between age and serum sCD4 levels (data not shown). Interestingly, significant correlation between serum sCD4 level and disease activity was found in the RA group but not in the SLE group. When the RA patients were divided into the low-disease activity (DAS28<3.2), moderate-disease activity (3.2≦DAS28≦5.1) and high-disease activity (DAS28>5.1) groups, a significant elevation in serum sCD4 levels was noted in the high-disease activity group compared with the low-disease activity group (Fig. 4B and Table 2). The moderate-disease activity group also has higher sCD4 levels than the low-disease activity group, but the difference is not statistically significant.

**Table 2:** Clinical characteristics and sCD4 levels of RA patients with low, moderate and high disease activity.

	Low disease Activity(DAS28≦3.2)	Moderate disease activity(3.2<DAS28≦5.1)	High disease activity(DAS28>5.1)
Disease duration (yrs)	4.53±5.41	4.77±6.08	9.40±8.55
ESR (mm/hr)	12.31±9.46	25.50±24.63	41.95±24.58
CRP (mg/L)	3.55±6.00	5.70±8.18	22.79±28.03
Steroid dosage (mg/day)	3.73±4.55	4.77±3.70	6.09±4.67
Biologic agents usage	Etanercept (3)	Etanercept (1) Adalimumab(2)	Etanercept (3) Adalimumab(7) Mabthera (1)
Soluble CD4 (ng/ml)Median (IQR 25^th^–75^th^)	0(0–0)	0(0–0.06475)	0.1839(0–1.016)

Data are expressed as means ± SD and median (interquartile range 25^th^–75^th^).

DAS28, 28-joint count Disease Activity Score; ESR, erythrocyte sedimentation rate; CRP, C-reactive protein; IQR, interquartile range.

To determine if there is a correlation between the sCD4 level and the outcome of RA patients, we assessed DAS28 of each RA patients again after 3 months and classified their clinical outcomes into no response, moderate response and good response based on the European League Against Rheumatism (EULAR) response criteria using DAS28. The ELISA data indicate the clinical outcomes are distinctively different between the patients with higher sCD4 levels (>0.125 ng/ml), which the cut off value is determined by the average background level of all normal subjects, and those with lower sCD4 levels (Table 3). Specifically, a significantly higher percentage of no responders is found among patients with the high serum sCD4 levels versus those with low sCD4 levels (94.4% versus 68.3%).

**Table 3:** Correlation between sCD4 levels in serum and clinical outcomes of RA patients after 3 months.

Outcome	Low level sCD4	High level sCD4	*P* value
No response	28 (68.3%)	17 (94.4%)	
Moderate response	10 (24.4%)	1 (5.6%)	0.034
Good response	3 (7.3%)	0 (0.0%)	

Data are expressed as number (%). All RA patients were assessed by DAS-28 again after 3 months and classified as no response, moderate response and good response according to EULAR response criteria. A low sCD4 level is defined as serum sCD4<0.125 ng/ml and a high sCD4 level is defined as serum sCD4≧0.125 ng/ml. Statistical significance was assessed by the trend analysis.

DAS28, 28-joint count Disease Activity Score; EULAR, European League Against Rheumatism.

## Discussion

Our data indicate that sCD4 generated from transfected CHO-K1 cells and primary CD4^+^ T cells is due to receptor shedding. This conclusion resonates with earlier reports showing that CD4 molecule can be cleaved by *Leishmaina*-derived peptidase gp63 and by bacteria exoproteases such as elastase and alkaline protease [31]. Most significantly, our results strongly suggest that endogenous cellular sheddases are also involved in the production of sCD4. While it is still unclear which specific sheddase is involved, the fact that the broad-spectrum metalloproteinase inhibitor, GM6001, efficiently inhibits CD4 shedding implicates members of MMP and/or ADAMs family as likely candidates. To further support this, it is noteworthy to know that gp63 is a MMP-like zinc-dependent endopeptidase, whose activity is inhibited by heavy metal ions and 1,10-phenanthroline, an inhibitor of metallopeptidases [31].

Previous studies have found evidence of overexpression of proteases by pathogens and cancer cells. This is thought to be beneficial for their own survival and growth by cleaving host cell-produced proteins in order to escape immune surveillance. Examples include acid protease secreted by bacteria for the proteolysis of IgA_1_ and C5a, cysteine and alkaline proteases secreted by parasites for cytolysis, and metalloproteinases secreted by cancer cells for tumor metastasis, invasion, immune escape and angiogensis [32], [33], [34]. Indeed, CD4 proteolysis by bacteria- and parasite-derived proteases might represent a general means for enhancing their infectivity and/or survival via inhibition of T cell activation and hence the adaptive immune response [31].

However, it is less clear about the role of sCD4 in chronic inflammatory diseases and HIV infection. As recombinant sCD4 has been shown to inhibit HIV infection, replication and syncytium formation selectively, it is possible that the increased sCD4 found in HIV-infected patients might serve as a negative feedback mechanism for the inhibition of further HIV infection [11], [35]. However, it is not known whether the endogenous sCD4 works similarly as recombinant sCD4. Similarly, the role of sCD4 in chronic inflammatory diseases might be a way to reduce CD4^+^ T cell activation. High levels of sCD4 might be able to compete with cell surface CD4 receptor for the binding of depleting and non-depleting CD4 mAbs, affecting the efficacy of these therapeutic agents. Alternatively, CD4 mAb binding *in vivo* might potentially enhance CD4 shedding (stripping) as reported in the case of Keliximab [36]. In this scenario, sCD4 could be considered as an anti-infectious and anti-inflammatory agent. On the other hand, sCD4 has also been shown to act as a chemoattractant for polymorphonuclear cells (PMNs) [35]. In this case, production of sCD4 in inflamed tissues would potentially lead to more immune cell infiltration resulting in secretion of additional MMPs by PMNs and possibly more sCD4, essentially amplifying tissue inflammation. Interestingly, MMPs are known to be overexpressed in many chronic inflammatory diseases including RA and SLE [34], [37], [38]. Hence, it is likely that MMPs produced by infiltrated immune cells in chronic inflammatory conditions actively shed sCD4, which in turn cause continued inflammation by attracting more leukocytes.

Previous studies have found increased levels of serum sCD4 in RA and SLE patients, indicating a relevant link to disease activity [13], [39], [40]. In our analysis of clinical serum samples, significantly higher levels of serum sCD4 were found in RA and SLE patients, but not in those with gout. More importantly, serum sCD4 levels were shown to be positively correlated with the disease activities of RA patients and patients with higher serum sCD4 levels are more refractory to present treatment within three months. These findings not only confirm previous studies [13], [20], but also seem to implicate sCD4 as an important parameter of disease severity/progression for certain chronic inflammatory diseases. The reason for the lack of correlation between sCD4 level and disease activities of SLE patients may be due to that the SLEDAI score gathered in this study is skewed to lower side and the heterogeneity of clinical manifestations in SLE patients also contributes to the difficulty in evaluating the relationship between soluble CD4 level and disease activities of SLE patients.

Hence, our results seem to suggest an association of sCD4 with a pro-inflammatory role in RA. The level of serum sCD4 has been considered as an indicator of T lymphocyte activation in various diseases [17], [41]. Higher disease activities in RA patients usually mean more T cell activation, hence increased levels of serum sCD4. Our present study suggests that sCD4 not only serves as a parameter of T cell activation, but also is associated with a potential pro-inflammatory role in RA. With this in mind, the relationship of sCD4 levels and MMPs production/activity in the chronic inflammatory diseases will be of great interest in the future.

## Supporting Information

Figure S1 **GM6001 inhibits constitutive and PMA-induced CD4 shedding in transfected CHO-K1 cells. (A, B)** CHO-K1 cells transiently transfected with the hCD4-myc construct were treated with DMSO (lane 1), PMA (100 nM, lane2), GM6001 (50 µM, lane 3) and PMA plus GM6001 (lane 4) for 2 days (48 hours). 20X concentrated conditioned medium (A) and cell lysates (B) were analyzed by Western blotting using with anti-CD4 and anti-myc mAbs as indicated. The blotted membrane was stained with Ponceau S solution to confirm the equal loading of conditioned medium and cell lysate. In addition, equal loading of cell lysate is checked by Western blotting with anti-actin mAb staining. Protein band intensity was measured by a densitometer and normalized against the corresponding β-actin band.
(TIF)

Figure S2 **GM6001 inhibits constitutive and PMA-induced CD4 shedding in transfected CHO-K1 cells. (A, B)** CHO-K1 cells transiently transfected with the hCD4-myc construct were treated with DMSO (lane 1), PMA (50 nM, lane2), GM6001 (50 µM, lane 3), PMA plus 50 µM GM6001 (lane 4) or PMA plus 100 µM GM6001 (lane 5) for 36 hours. 20X concentrated conditioned medium (A) and cell lysates (B) were analyzed by Western blotting using with anti-CD4 and anti-myc mAbs as indicated. The blotted membrane was stained with Ponceau S solution to confirm the equal loading of conditioned medium and cell lysate. In addition, equal loading of cell lysate is checked by Western blotting with anti-actin mAb staining. Protein band intensity was measured by a densitometer and normalized against the corresponding β-actin band.
(TIF)
