# Supplementary figures and images for: Increased Soluble CD4 in Serum of Rheumatoid Arthritis Patients Is Generated by Matrix Metalloproteinase (MMP)-Like Proteinases
Source: PLoS One. 2013 May 21;8(5):e63963. doi: 10.1371/journal.pone.0063963 (PMC3660307; doi:10.1371/journal.pone.0063963)

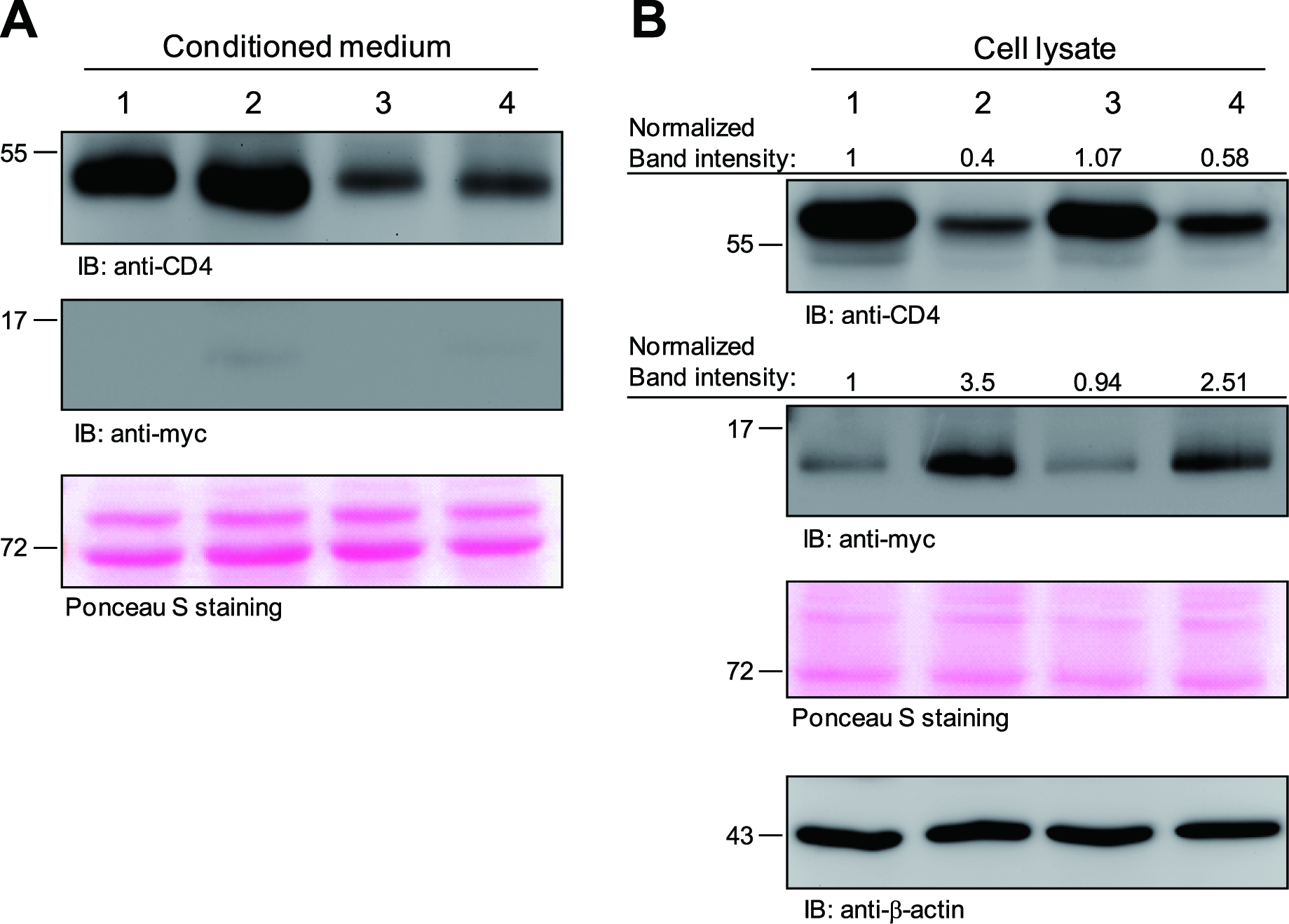

Supplement: Figure S1 — GM6001 inhibits constitutive and PMA-induced CD4 shedding in transfected CHO-K1 cells. (A, B) CHO-K1 cells transiently transfected with the hCD4-myc construct were treated with DMSO (lane 1), PMA (100 nM, lane2), GM6001 (50 µM, lane 3) and PMA plus GM6001 (lane 4) for 2 days (48 hours). 20X concentrated conditioned medium (A) and cell lysates (B) were analyzed by Western blotting using with anti-CD4 and anti-myc mAbs as indicated. The blotted membrane was stained with Ponceau S solution to confirm the equal loading of conditioned medium and cell lysate. In addition, equal loading of cell lysate is checked by Western blotting with anti-actin mAb staining. Protein band intensity was measured by a densitometer and normalized against the corresponding β-actin band. (TIF) [file pone.0063963.s001.tif]

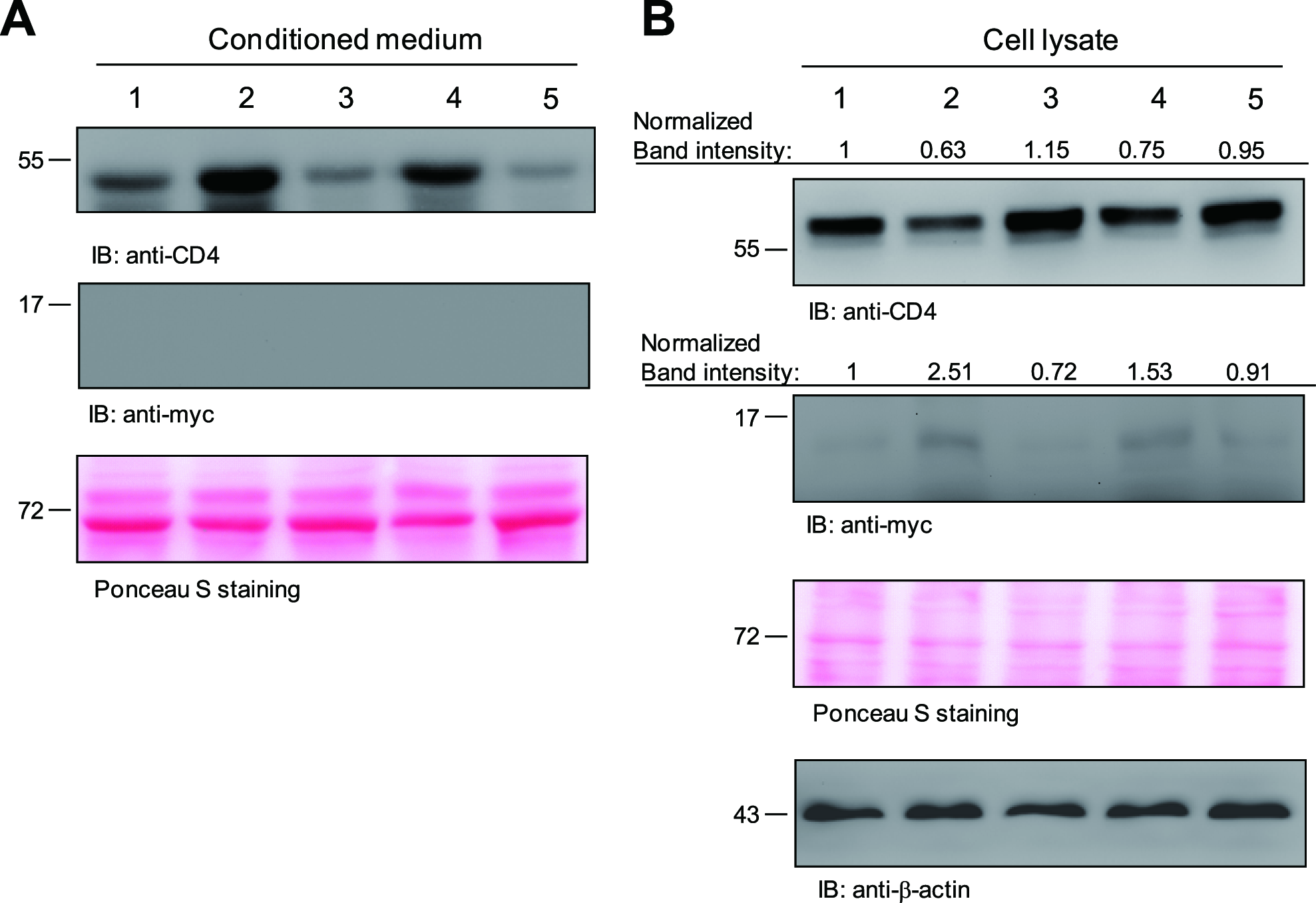

Supplement: Figure S2 — GM6001 inhibits constitutive and PMA-induced CD4 shedding in transfected CHO-K1 cells. (A, B) CHO-K1 cells transiently transfected with the hCD4-myc construct were treated with DMSO (lane 1), PMA (50 nM, lane2), GM6001 (50 µM, lane 3), PMA plus 50 µM GM6001 (lane 4) or PMA plus 100 µM GM6001 (lane 5) for 36 hours. 20X concentrated conditioned medium (A) and cell lysates (B) were analyzed by Western blotting using with anti-CD4 and anti-myc mAbs as indicated. The blotted membrane was stained with Ponceau S solution to confirm the equal loading of conditioned medium and cell lysate. In addition, equal loading of cell lysate is checked by Western blotting with anti-actin mAb staining. Protein band intensity was measured by a densitometer and normalized against the corresponding β-actin band. (TIF) [file pone.0063963.s002.tif]
